# High-dose intravenous vitamin C reduce C-reactive protein levels, fluid retention, and APACHE II scores in patients with moderately severe acute pancreatitis: a prospective, randomized, double-blinded, placebo-controlled study

**DOI:** 10.1186/s13613-025-01437-z

**Published:** 2025-03-17

**Authors:** Bing Zhao, Wenwu Sun, Yihui Wang, Li Ma, Menglu Gui, Jiaoyan Li, Xianxian Yu, Xing Qi, Ning Ning, Silei Sun, Mengjiao Li, Yi Yao, Tongtian Ni, Juan He, Zhitao Yang, Ying Chen, Huiqiu Sheng, Meihua Shen, Jian Li, Jun Huang, Enqiang Mao

**Affiliations:** 1 Department of Emergency, Ruijin Hospital, Shanghai Jiao Tong University School of Medicine, Shanghai, 200025 P. R. China; 2 Department of Pharmacy, Ruijin Hospital, Shanghai Jiao Tong University School of Medicine, Shanghai, 200025 P. R. China; 3 National Center for Translational Medicine (Shanghai) SHU Branch, Shanghai University, Shanghai, PR China; 4 Department of Critical Care Unit, Shanghai Provincial CorpsHospital, Chinese People’s Armed Police Forces, Shanghai, PR China; 5 Clinical Research Center, Ruijin Hospital, Shanghai Jiao Tong University School of Medicine, Shanghai, 200025 P. R. China; 6 Department of Cardiovascular Medicine, State Key Laboratory of Medical Genomics, Shanghai Key Laboratory of Hypertension, Shanghai Institute of Hypertension, Ruijin Hospital, Shanghai Jiao Tong University School of Medicine, Shanghai, 200025 P. R. China; 7 Department of Emergency Medicine, Daping Hospital, State Key Laboratory of Trauma and Chemical Poisoning, Army Medical University, Chongqing, 400042 China

**Keywords:** Severe acute pancreatitis, Vitamin C, Randomized controlled trial, Mortality, C-reactive protein, Fluid retention

## Abstract

**Background:**

The aim of this study was to investigate whether high-dose intravenous vitamin C (HDIVC) could decrease the mortality rate within 28 days among patients moderately severe acute pancreatitis (MSAP) and severe acute pancreatitis (SAP).

**Methods:**

In this randomized, placebo-controlled trial, patients diagnosed with predicted MSAP or SAP within 72 h of symptom onset were enrolled to receive either a vitamin C infusion (200 mg/kg/24 h) or a matched placebo for 7 days. The primary outcome was 28-day mortality.

**Results:**

212 adults including 155 MSAP and 57 SAP were enrolled from September 2019 to June 2023. The trial was terminated prematurely due to a lower than expected 28-day mortality rate which showed no difference between the HDIVC and Control group (3/109 vs. 4/103, unadjusted OR: 0.70, 95% CI, 0.15–3.21, *p* = 0.647). Among patients with MSAP, the HDIVC group exhibited a more pronounced reduction in C-reactive protein levels compared to the Control group (Day0 to Day3, median 72 mg/L vs. 46 mg/L, *p* = 0.003; Day0 to Day7, median 168 mg/L vs. 121 mg/L, *p* = 0.013); The volume of fluid retention was lower in the HDIVC group compared to the Control group (Day0-Day1, median 676.5 ml vs. 1130 ml, *P* = 0.04; Day0-Day2, median 511 ml vs. 1290 ml, *P* = 0.02; Day0-Day3, median 692 ml vs. 1534 ml, *P* = 0.04). The APACHE II scores reduction from Day0 to Day7 was significantly greater in the HDIVC group in APACHE II scores (median change of 3 vs. 2, *P* = 0.01). No significant difference was observed among patients with SAP.

**Conclusion:**

HDIVC did not significantly reduce 28-day mortality in MSAP and SAP patients. While it showed potential benefits in reducing CRP, fluid retention, and APACHE II scores in MSAP patients, these effects may not be directly related to the study drug, and no similar changes were observed in SAP patients.

**Trial registration:**

ChiCTR.org.cn, ChiCTR1900022022. Registered March 21 2019, https//www.chictr.org.cn/showproj.html?proj=37,106.

**Supplementary Information:**

The online version contains supplementary material available at 10.1186/s13613-025-01437-z.

## Background

Acute pancreatitis (AP) is a prevalent gastrointestinal disorder, with an annual incidence of 34 cases per 100,000 individuals [1]. Approximately 20% of patients eventually progress to moderately severe AP (MSAP) or severe AP (SAP). The severity of AP is marked by an uncontrolled systemic inflammatory response, accumulation of fluid in the third interstitial space, necrosis of pancreatic or peripancreatic tissue, and transient or persistent organ failure [2]. Despite significant improvements in treatment methods over recent decades, the overall mortality rate among SAP patients remains high, ranging from 20 to 40% [3, 4].

Uncontrolled oxidative stress triggers an uncontrolled systemic inflammatory response, ultimately resulting in endothelial dysfunction and multiple organ dysfunctions (MODS) [5].Furthermore, early MODS serves as an independent risk factor for secondary pancreatic infections among patients who survive the initial stage [6]. Consequently, the timely elimination of excessive oxidative free radicals is considered a crucial aspect of enhancing patient prognosis. Vitamin C, a vital antioxidant present in plasma that effectively neutralizes reactive oxygen species (ROS) [7], has demonstrated the ability to reduce inflammation and protect the endothelial barrier. Our preclinical study demonstrated a significant association between vitamin C supplementation and improved prognosis, including reduced inflammatory markers, mortality rates, and pancreatic necrosis in a rat model of severe acute pancreatitis (SAP) [8, 9]. However, despite four clinical trials examining the therapeutic effects of vitamin C in acute pancreatitis (AP), a definitive conclusion remains elusive, possibly due to limited sample sizes, insufficient vitamin C dosing, or relatively mild disease severity [10–13].

A recently developed treatment known as high dose intravenous vitamin C (HDIVC) has demonstrated promising results in treating various critical conditions, including sepsis, ARDS, and severe cases of COVID-19 [14]. For instance, the CITRIS-ALI trial demonstrated that HDIVC significantly reduced 28-day mortality among patients with sepsis-associated acute lung injury [15]. In this study, we aimed to investigate whether HDIVC (200 mg/kg/24 h for 7 days) could reduce the risk of death within 28 days among MSAP and SAP patients. To achieve this, we conducted a prospective, randomized, double-blinded, and placebo-controlled trial.

## Methods

### Study design and participants

This randomized, placebo-controlled trial was conducted at a single center and was approved by the institutional ethics board of Ruijin Hospital, Shanghai Jiao Tong University School of Medicine (registration number: 2019-90). It was registered with ChiCTR (ChiCTR1900022022). The protocol for this trial has been previously published [16]. Prior to randomization, written informed consent was obtained from each participant or their first-degree relative. The trial was undertaken from September 2019 to December 2023, with the first participant being enrolled on September 11, 2019.

### Participants

Participants were recruited from the Department of Emergency, General Surgery, and Gastroenterology at Ruijin Hospital, Shanghai Jiao Tong University School of Medicine. Eligible participants were adult patients aged between 18 and 75 years, with AP who met the diagnostic criteria for MSAP and SAP as outlined in the 2012 revised Atlanta guideline [17]. Specially, MAP (mild acute pancreatitis) is characterized by the absence of organ failure and local or systemic complications. MSAP is diagnosed by transient organ failure (that is, organ failure resolving within 48 h) and/or the presence of local or systemic complications. SAP is diagnosed by the presence of persistent organ failure (that is, organ failure lasting more than 48 h). Additional criteria for inclusion were a time interval of 72 h or less from symptom onset to enrollment, and proficiency in reading and writing Chinese. The diagnosis of MSAP or SAP was based on predicted severity at enrollment, as it was challenging to determine whether organ failure would persist beyond 48 h at that stage. Individuals with AP caused by malignant tumor or ERCP, who are pregnant or lactating, as well as those with poorly controlled chronic organ failure, including (1) chronic cardiovascular dysfunction requiring long-term mechanical hemodynamic support or inotropic support; (2) chronic obstructive pulmonary disease necessitating home oxygen therapy; (3) chronic hepatic dysfunction classified as Child‒Pugh C; and (4) chronic renal disease with an eGFR less than 60 mL/min/1.73 m2 or serum creatinine exceeding 150µmol/L, were excluded from the study. Additionally, patients with sepsis prior to admission, those with autoimmune diseases or a persistent immunosuppressive state (such as unresolved malignant tumor, post-transplantation status, prolonged use of immunosuppressive agents or hormones, AIDS, etc.), those with kidney stones, those allergic to the experimental drugs, those implanted with a special device incompatible with imaging examination, or those participating in other clinical trials were also excluded.

### Randomization and masking

Patients who met the eligibility criteria were randomized 1:1 using block randomization to receive treatment with HDIVC (200 mg/kg/24 h) or placebo (saline) for 7 days, and the block size was accessible to those who performed the randomization. A randomization sequence was generated using a computer-generated randomized sequence and sealed in envelopes by a statistician unrelated to the trial. The randomization information will be blinded to all the participants in the trials except for the unblinded drug preparing nurse (UDPN). The UDPN unsealed the envelope and disposed of vitamin C or saline in a 50 ml syringe according to the grouping information inside the envelope. The UDPN gave opaque syringes to the therapy nurse independent of the following medical process. Participants and the research team were blinded to the allocation.

### Procedures

The participants accepted standard treatment for acute pancreatitis, encompassing intensive care, fluid therapy, proton pump inhibitors, nutritional support, and antibiotics in cases of suspected infection. A subgroup of patients who met the indications for fluid resuscitation were defined as follows: heart rate ≥ 120 beats/min; mean arterial pressure ≥ 85 mmHg or ≤ 60 mmHg; blood lactate concentration ≥ 2 mmol/L; urine output ≤ 0.5 mL·kg-1·h-1; and hematocrit level ≥ 44%, if three or more of the listed criteria were met, fluid resuscitation was initiated. These patients underwent blood volume expansion according to the protocol of controlled fluid resuscitation [18]. If the mean arterial pressure (MAP) is less than 60 mmHg, it must be increased to above 60 mmHg within 30 min using a vasopressor and rapid infusion. After this initial phase, the infusion rate should be adjusted to an optimal level of 5–10 mL/kg/hour. During blood volume expansion, indicators should be assessed every 4 h. If two or more of the following criteria are met—heart rate less than 120 beats per minute, MAP between 65 and 85 mmHg, normal urine output, or hematocrit (HCT) levels between 30% and 35%—the blood volume expansion should be discontinued. In the HDIVC cohort, patients received a dose of 200 mg/kg/24 h of vitamin C mixed in a 50 ml solution of normal saline, administered intravenously through a central venous line. This infusion was administered separately from other medications and continued for seven consecutive days following randomization. Conversely, the placebo group received an equal volume of normal saline.

The patients were enrolled within one hours after admission. Initial variables were collected at enrollment, and monitoring variables were recorded each day before administering the study agent. The study agents were administered daily at 6 am for a maximum of seven days, or until the patient’s discharge from the ICU or death, whichever occurred first. The first day of study agent administration was defined as Day 0.

### Outcomes

The primary outcome of the study was 28-day all-cause mortality. Prespecified secondary outcomes included: (1) changes in inflammatory biomarkers, such as plasma C-reactive protein (CRP) levels from Day 0 to Day 3 and Day 7, ratio of patients with systemic inflammatory response syndrome (SIRS) on Day 3 and Day 7; (2) assessment of fluid retention, defined as the total input volume of intravenous fluid and enteral nutrition minus the output volume of urine and drainage; (3) evaluation of the risk of organ failure lasting for more than 48 h; (4) changes in Sequential Organ Failure Assessment (SOFA) scores, Acute Physiology and Chronic Health Evaluation (APACHE) II scores, modified Marshall score, and Balthazar score from Day 0 to Day 3 and Day 7; (5) assessment of the duration of organ support, including mechanical ventilation and renal replacement therapy within 3 and 7 days; (6) evaluation of 90-day all-cause mortality. SIRS is defined by the presence of at least two of the following criteria: fever or hypothermia, tachycardia, tachypnea, and leukocyte abnormalities.

Adverse effects (AEs) associated with vitamin C, such as crystals in the urine, unexplained respiratory failure, thromboembolic disease, arrhythmias, delirium, and anemia, were monitored and recorded. Any unexpected serious adverse effects considered to be related to vitamin C were promptly reported to the principal investigator, and the administration of the medication was discontinued immediately upon confirmation.

### Statistical analysis

The sample size calculation is based on the primary outcomes of 28-day mortality in patients with moderately severe and severe acute pancreatitis. Based on the data of previously published retrospective study in our center [19], it was estimated that the mortality rate within 28 days would be 35%. We anticipated that the 28-day mortality rate would be 10% lower in the HDIVC group than in the placebo group. We calculated that the inclusion of 418 patients (209 patients per arm) would provide the trial with 80% power to detect a difference of 10% in the 28-day mortality rate at a two-sided significance level of 5% by PASS 11.0 software (NCSS, Kaysville, UT), taking into accounting for 10% of dropout.

On May, 2023, an interim analysis was performed after 212 participants were enrolled, we observed that the 28-day mortality rate among patients with MSAP and SAP was 3% (7/212), significantly lower than the anticipated level. For ethical reasons, the independent data monitoring committee recommended to terminate the enrolling of patients, early analyze and publish the finding (refer to Supplementary material 1 for further details). The database was locked on June 19, 2023, at which time 212 patients, encompassing 155 cases of MSAP and 57 of SAP, had been enrolled. The findings from this analysis are presented here.

Categorical data will be described as the frequency or proportion. Continuous variables will be described using median and interquartile range (IQR) or the mean and standard deviation as appropriate. Categorical data were compared using the chi-square test or Fisher’s exact test. Continuous variables were compared using the t test for normally distributed variables or the Wilcoxon rank-sum test for non-normally distributed variables. The primary analysis was conducted using the intention-to-treat (ITT) approach. A logistic regression model was used to compare the number of deaths between groups, and the odds ratio (OR) was calculated. In the adjusted analysis, a linear mixed model was used for longitudinal data, with individuals adjusted as a random effect variable. A two-sided P value < 0.05 was considered to indicate statistical significance. The P values for multiple comparisons of the secondary end points were not adjusted, so the analyses of secondary end points should be interpreted as exploratory. All the statistical analyses were performed using SPSS (version 19.0) and R software (version 4.2.1).

## Results

### Patient enrollment and characteristics

**Fig. 1:**
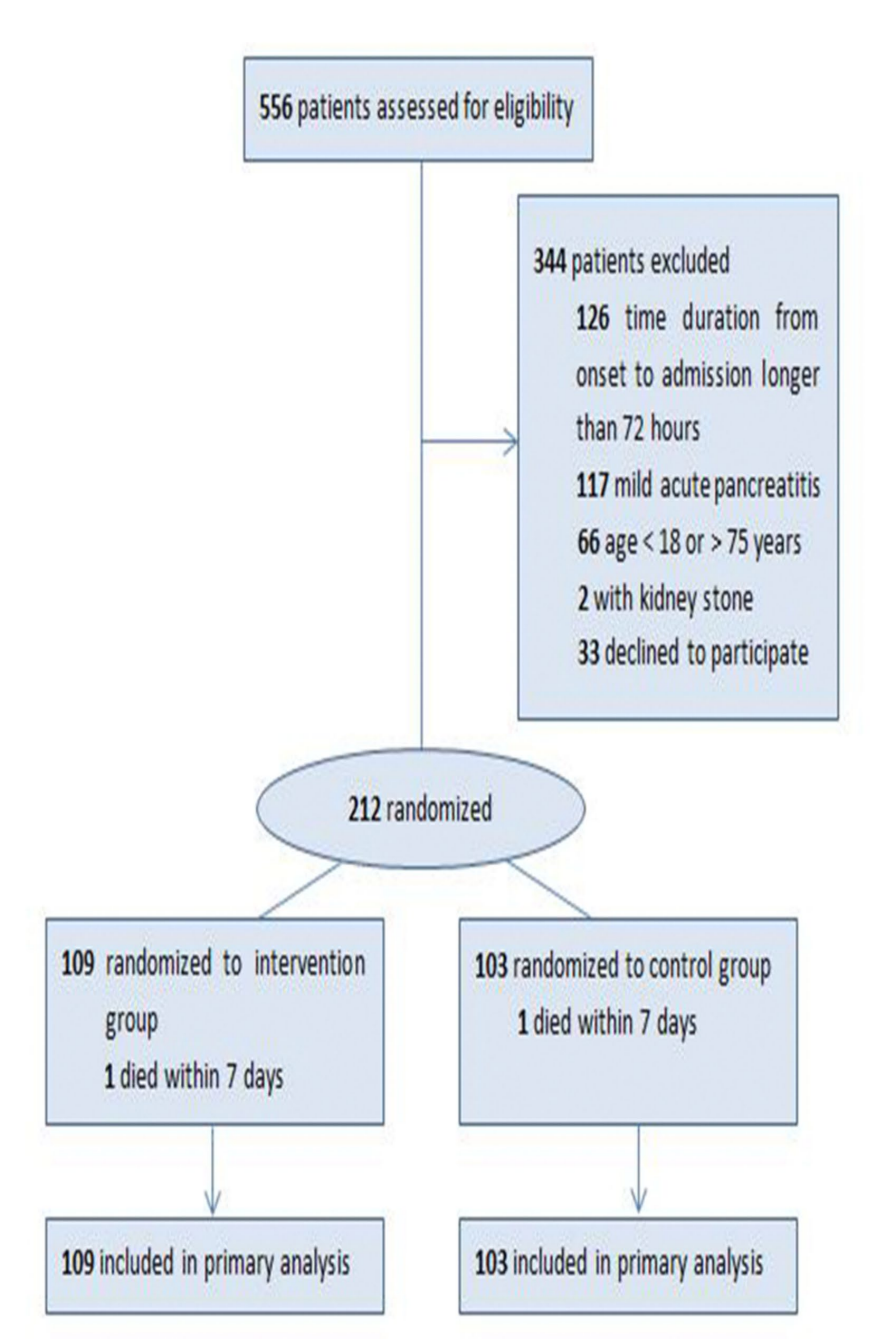
Screening flowchart

Between September 2019 and May 2023, a total of 556 patients underwent screening, of which 212 met the inclusion criteria and were randomized for the study (Fig. 1). The primary reasons for exclusion included a duration of more than 72 h from onset to admission (*n* = 126) and a diagnosis of mild acute pancreatitis (*n* = 117). Following randomization, 212 patients were included in the intention-to-treat (ITT) analyses, with 109 patients allocated to the HDIVC group and 103 patients allocated to the Control group. 23 of 109 patients in the HDIVC group and 28 of 103 patients in the Control group were transferred from other hospital and their pre-enrollment treatment are comparable (Supplementary Table 1). It is important to note that one patient from each group unfortunately passed away within 7 days. However, a total of 210 patients successfully completed the 7-day intervention and the subsequent 90-day follow-up period.

We examined the baseline characteristics of the enrolled patients in the HDIVC and control groups. The demographic data showed no significant differences between the two groups in terms of age, gender distribution, body mass index, time from onset to admission, medical history, pathogenesis, vital signs, severity, and the need for various interventions between the HDIVC and control groups (Table 1). Patients with SAP accounted for 24.8% (*n* = 27) in the HDIVC group and 29.1% (*n* = 30) in the placebo group. The specific diagnostic criteria for MSAP, including local complications and organ failure, are provided in Supplementary Table 2.

**Table 1:** Patients characteristic

vVariables	HDIVC^h^ (*n* = 109)	Control (*n* = 103)	*P*-value
Demographic data			
Age, median (IQR^a^), y	41 (33, 50)	44 (35, 55)	0.160
Male (%)	71(65.1)	69 (67.0)	0.776
BMI, median (IQR)	25.8 (23.4, 29.1)	26.1 (22.8, 29)	0.720
Amylase, median (IQR), U/L	502 (278, 1196)	633 (352.8, 1459)	0.112
Time from onset to randomization, median (IQR), d	2 (1, 2)	2 (1, 3)	0.179
Medical history, No (%)			
Hypertension	35 (32.1)	34 (33.0)	0.889
Diabetes	34 (31.2)	27 (26.2)	0.423
Hyperlipidemia	45 (41.28)	29 (28.16)	0.060
Etiologies, No (%)			
Biliary	39 (35.8)	43 (41.7)	0.373
Hypertriglyceridemic	61 (56.0)	52 (50.5)	0.424
Alcoholic	5 (4.6)	5 (4.9)	1
Hypercalcemic	1 (0.9)	1 (1.0)	1
Other	3 (2.8)	2 (1.9)	1
Vital signs on Day0, median (IQR)			
Temperature (℃)	37.1 (36.8, 38.1)	37.2(36.9, 37.8)	0.840
Systolic blood pressure (mmHg)	138 (128, 151)	135 (121, 150)	0.126
Diastolic blood pressure (mmHg)	82 (73, 91)	80 (73, 92)	0.716
Pulse rate (times per minute)	114 (98, 127)	113 (94, 126)	0.813
Breath rate (times per minute)	26 (21, 31)	25 (20, 30)	0.7
APFC^b^ on Day0, No (%)	65 (59.63)	60 (58.25)	0.889
ANC^c^ on Day0, No (%)	44 (40.37)	43 (41.75)	
Severity on Day0, No (%)			0.475
MSAP^d^	82 (75.2)	73 (70.9)	
SAP^e^	27 (24.8)	30 (29.1)	
MV^f^ on Day0, No (%)	18 (16.5)	24 (23.3)	0.215
RRT^g^ on Day 0, No (%)	13 (11.9)	14 (13.6)	0.716
Vasoactive drug on Day 0, No (%)	5 (4.6)	9 (8.7)	0.347
Patients need Fluid resuscitation on Day 0, No (%)	70 (64.2)	63 (61.2)	0.646

(a) IQR, interquartile range; BMI, body mass index; (b) APFC, acute peripancreatic fluid collection; (c) ANC, acute necrosis collection; (d) MSAP: moderately severe acute pancreatitis; (e) SAP: severe acute pancreatitis; (f) MV, mechanical ventilation; (g) RRT, renal replacement treatment; (h) HDIVC, high dose intravenous vitamin C.

### Primary outcomes

**Fig. 2:**
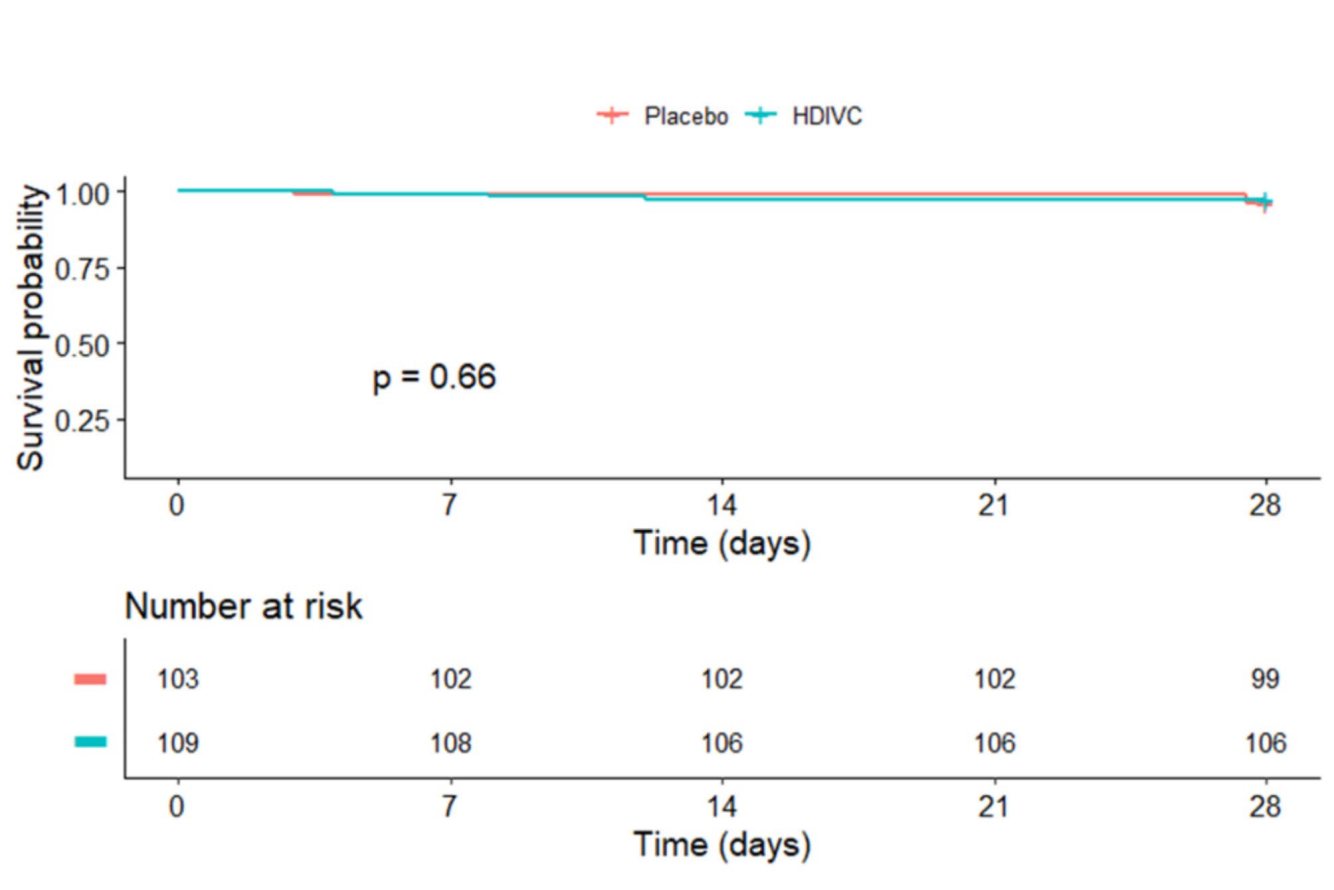
Kaplan–Meier curve between two groups

There was no statistically significant difference observed in the 28-day all-cause mortality rates between the HDIVC group and the control group (Fig. 2). In the HDIVC group, there were 3 deaths (2.8%), while the placebo group had 4 deaths (3.9%). The unadjusted odds ratio (OR) for death in the HDIVC group at 28 days was 0.70 (95% CI, 0.15–3.21; *p* = 0.647).

### Secondary outcomes

As indicated in Table 2, there were no significant differences observed in CRP levels on Day3 and Day7 between the HDIVC and Control groups. However, the reduction in CRP levels was notably more pronounced in the HDIVC group compared to the Control group from Day0 to Day3 (median 72 mg/L vs. 46 mg/L, *p* = 0.003) and from Day0 to Day7 (median 168 mg/L vs. 121 mg/L, *p* = 0.013). In the subgroup analysis, the decrease in CRP levels from Day0 to Day3 (median 74 mg/L vs. 43 mg/L, *p* = 0.0003) and Day7 (median 177 mg/L vs. 131 mg/L, *p* = 0.013) was more evident in the HDIVC group than in the Control group among patients with MSAP, but not in those with SAP. The proportion of CRP decline from Day0 to Day3 but not Day0 to Day7 also differed between the two groups (Supplementary Table 3). The occurrence and duration of systemic inflammatory response syndrome (SIRS) serve as additional indicators of the inflammatory response, and no significance was found among the whole cohort, MSAP and SAP patients.

**Table 2:** Effect of HDIVC on inflammatory response

Variables	Time Point	*n*	HDIVC^f^	*n*	Control	*P*-value	
**CRP**^**a**^**of whole cohort**,** median (IQR**^**b**^**)**,** mg/L**	Day0	109	272 (203, 315)	103	246 (152, 299)	0.03	
	Day3	109	178 (124,256)	103	201(133, 255)	0.28	
	Day7	109	82.5 (29.8, 143.8)	103	73.9 (22.5,135.5)	0.29	
**CRP of MSAP**^**c**^, **median (IQR)**,** mg/L**	Day0	82	270 (186.3,318.3)	73	221 (147, 285)	0.02	
	Day3	82	176.5 (118.8, 228.5)	73	201 (132.5, 260.5)	0.11	
	Day7	80	67(25.5, 120.5)	73	51.5 (18.25, 117.8)	0.36	
**CRP of SAP**^**d**^, **median (IQR)**,** mg/L**	Day0	27	286 (206, 309)	30	269 (173.3, 328)	0.63	
	Day3	27	198 (125, 291)	30	198.5 (125, 291)	0.75	
	Day7	26	136 (75.5)	29	142 (72.3, 220.5)	0.89	
**Delta CRP of whole cohort**,** median (IQR)**,** mg/L**	Day0-Day3	109	72 (22.5, 120.5)	103	46 (-29, 97)	0.0029*	
	Day0-Day7	109	168 (101, 233.3)	103	123 (43, 199)	0.0097*	
**Delta CRP of MSAP**,** median (IQR)**,** mg/L**,	Day0-Day3	82	74 (17,75, 115.8)	73	43 (-49, 94.5)	0.0003*	
	Day0-Day7	81	177 (112.5, 239.5)	73	131 (54, 215.5)	0.013*	
**Delta CRP of SAP**,** median (IQR)**,** mg/L**	Day0-Day3	27	56 (25, 138)	30	60.5 (14, 100.8)	0.88	
	Day0-Day7	27	119.1 (119.3)	30	104.8 (93.65)	0.61	
**Patients with SIRS**^**e**^**of whole cohort**,** No (%)**	Day0	109	91 (83.5)	103	86 (83.5)	0.99	
	Day3	109	68 (62.4)	103	61 (59.2)	0.637	
	Day7	109	24 (22.2)	103	33 (32.4)	0.099	
**Patients with SIRS of MSAP**,** No (%)**	Day0	82	64 (78.05)	73	58 (79.45)	> 0.83	
	Day3	82	31 (48.44)	73	27 (47.37)	> 0.9	
	Day7	82	8 (12.31)	73	7 (12.28)	> 0.9	
**Patients with SIRS of SAP**,** No (%)**	Day0	27	26 (96.3)	30	28 (90.32)	> 0.9	
	Day3	27	24(92.31)	30	26(89.66)	> 0.9	
	Day7	27	18 (66.67)	30	26 (86.67)	0.114	
**SIRS Duration of whole cohort**,** d**,** median (IQR)**	Day0-Day7	109	3 (2, 7)	103	3 (2, 7)	0.503	
**SIRS Duration of MSAP**,	Day0-Day7	82	2 (1, 7)	56	2 (1,7)	0.6819	
**SIRS Duration of SAP**,** median (IQR)**,** d**	Day0-Day7	27	5 (7, 7)	28	7 (7, 7)	0.0551	

(a) CRP, C-reactive protein; (b) IQR, interquartile range; (c) MSAP, moderately severe acute pancreatitis; (d) SAP, severe acute pancreatitis; (e) SIRS, systemic inflammatory response syndrome; (f) HDIVC, high dose intravenous vitamin C. *: *P* < 0.05

As shown in Table 3, in the whole cohort, there was no significant difference in fluid retention volume between the two groups on Day0 (first 24 h after enrollment). However, a decrease in fluid retention was observed during Day0-Day1 (first 48 h after enrollment) with a median of 889 mL vs. 1340 mL, *p* = 0.03. Subsequently, there was a significant decrease in fluid retention during Day0-Day2 (first 72 h after enrollment) with a median of 885 mL vs. 1511 mL, *p* = 0.02, and Day0-Day 3 (first 96 h after enrollment) with a median of 949 mL vs. 1656 mL, *p* = 0.02. Subgroup analysis revealed a similar trend in patients with MSAP (Day0, median 859.5 ml vs. 870.5 ml, *P* = 0.34; Day0-Day1, median 676.5 ml vs. 1130 ml, *P* = 0.04; Day0-Day2, median 511 ml vs. 1290 ml, *P* = 0.02; Day0-Day3, median 692 ml vs. 1534 ml, *P* = 0.04). No significant difference was showed in SAP patients.

**Table 3:** Effect of HDIVC on fluid retention

Variables, median (IQR^a^), ml	Time Point	*n*	HDIVC^d^	*n*	Control	*P*-value
**Fluid retention in whole cohort**	Day0	103	935 (365, 2035)	98	1043 (422.5, 2024)	0.32
	Day0-Day1	103	889 (-143, 2450)	98	1350 (287.3, 2937)	0.03*
	Day0-Day2	103	885 (-300, 2656)	98	1511 (200, 3224)	0.02*
	Day0-Day3	103	949 (-380, 2800)	98	1656 (110.5, 3886)	0.02*
**Fluid retention in MSAP** ^**b**^	Day0	78	859.5 (84.8, 1805)	72	870.5 (252.5, 1639)	0.34
	Day0-Day1	76	676.5(-495.8,1719)	72	1130 (163.5, 1833)	0.04*
	Day0-Day2	78	511 (-363.8, 1750)	72	1290 (94.8, 2442)	0.02*
	Day0-Day3	76	692 (-648.5, 1866)	72	1534 (-139, 2739)	0.04*
**Fluid retention in SAP** ^**c**^	Day0	27	1900 (550, 2600)	27	2660 (995, 4205)	0.258
	Day0-Day1	27	2455 (610, 4840)	27	4615 (800, 7567)	0.13
	Day0-Day2	27	2937(-300, 4284)	27	3891 (1085,7913)	0.16
	Day0-Day3	27	2975 (-75, 3873)	27	5122 (1042,7867)	0.11

(a) IQR, interquartile range; (b) MSAP, moderately severe acute pancreatitis; (c) SAP, severe acute pancreatitis; (d) HDIVC, high dose intravenous vitamin C

Fluid retention is calculated as the total input, including intravenous and enteral fluids, minus the total output, including urine and drainage. Day 0 refers to the first 24 h after enrollment, Day 0-Day 1 refers to the first 48 h after enrollment, Day 0-Day 2 refers to the first 72 h after enrollment, and Day 0-Day 3 refers to the first 96 h after enrollment. *: *P* < 0.05

Determining whether organ failure, including the respiratory, renal, and cardiovasular system, persists beyond 48 h is a critical question, as this threshold can delineate SAP from MSAP. Table 4 illustrates that the incidence of organ failure on admission was 52 cases in the HDIVC group and 45 in the Control group. Among whom, the number of patients with followed persistent organ failure (lasting more than 48 h) was 27 in the HDIVC group and 30 in the Control group, with no statistically significant difference (*P* = 0.125). The number of SAP patients accepted MV or RRT was similar between the HDIVC and the Control group. The SOFA score did not demonstrate a significant difference across the entire cohort or within the MSAP subgroup. However, within the SAP subgroup, the SOFA score was significantly lower in the HDIVC group on Day 0 (*P* = 0.002) and Day 3 (*P* = 0.02). Additionally, the change in SOFA score revealed no significant differences between the two groups. The Acute Physiology and Chronic Health Evaluation (APACHE II) scores were comparable on Day0, Day3, and Day7 between the two groups, both in the entire cohort and within the MSAP subgroup. Intriguingly, the overall change in APACHE II scores from Day0 to Day7 was significantly greater in the HDIVC group than in the Control group for the entire cohort (median change of 4 vs. 2, *P* = 0.03). Subgroup analysis revealed that this trend was particularly pronounced among MSAP patients, with a significantly greater change in APACHE II scores in the HDIVC group (median change of 3 vs. 2, *P* = 0.01). However, this difference was not observed in SAP patients. We further analyzed the components of the APACHE II score. The change in the Acute Physiology Score (APS) from Day 0 to Day 7 was consistent with the overall APACHE II score in the whole cohort (Supplementary Table 4) and the MSAP subgroup (Supplementary Table 5) but not in the SAP subgroup (Supplementary Table 6). No significant difference was observed in the change of Chronic Health Score (CHS). The APACHE II score, excluding the age component (APS plus CHS), also mirrored the overall APACHE II score. Additionally, the other two severity indicators, namely the Marshall and Balthazar scores, did not exhibit any significant differences. The dynamics of the scores on Day0, Day3 and Day3 were showed in Supplementary Fig. 1. We also showed 90-day mortality was similar between the two group (Supplementary Table 7).

**Table 4:** Effect of HDIVC on organ function and severity scores

Variable, median (IQR^a^), ml	time point	*n*	HDIVC^f^	*n*	Control	*P*-value	
**Patients with organ failure on admission**,** No**	Day0	52	52	45	45		
**Patients with organ failure lasting > 48 h**,** No (%)**	Day0-Day7	27	27 (51.92)	30	30 (66.67)	0.125	
**Patients with organ failure lasting < 48 h**,** No (%)**	Day0-Day7	25	25 (48.08)	15	15 (33.33)		
**RRT**^**b**^**free duration in SAP patients**,** median (IQR)**	Day0-Day3	27	72 (52, 72)	30	72 (30,72)	0.737	
	Day0-Day7	27	168 (148,168)	30	168 (126, 168)	0.737	
**MV**^**c**^**free duration in SAP patients**,** median (IQR)**,** hours**	Day0-Day3	27	0 (0,72)	30	0 (0,0)	0.25	
	Day0-Day7	27	72 (0,168)	30	15 (0,88)	0.179	
**SOFA score in whole cohort**,** median (IQR)**	Day0	109	2 (0, 3)	103	2 (1, 4)	0.15	
	Day3	109	2 (0, 4)	103	2 (0, 3)	0.13	
	Day7	109	0 (0, 2)	103	0 (0, 2.25)	0.19	
**SOFA score in MSAP**^**d**^, **median (IQR)**	Day0	82	1 (0, 3)	73	2 (1, 4)	0.586	
	Day3	82	1 (0, 2)	73	1 (0, 2)	0.661	
	Day7	82	0 (0, 1)	73	0 (0, 0)	0.903	
**SOFA score in SAP**^**e**^, **median (IQR)**	Day0	27	4 (3, 5)	30	6 (4, 8)	0.002	
	Day3	27	4 (3, 6)	30	6 (4, 9)	0.02	
	Day7	27	2 (0.75, 4.5)	30	4 (1, 7)	0.072	
**Delta SOFA score in whole cohort**,** median (IQR)**	Day0-Day3	109	0 (-1, 1)	103	0 (-1, 1)	0.63	
	Day0-Day7	109	1 (0, 2)	103	1 (0, 2)	0.93	
**Delta SOFA score MSAP**,** median (IQR)**	Day0-Day3	82	0 (-1, 1)	73	0 (-1, 1)	0.63	
	Day0-Day7	82	1 (0, 2)	73	1 (0, 2)	0.93	
**Delta SOFA score SAP**,** median (IQR)**	Day0-Day3	27	0 (-1, 1)	30	0 (-1, 1)	0.63	
	Day0-Day7	27	1 (0, 2)	30	1 (0, 2)	0.93	
**APACHEII score in whole cohort**,** median (IQR)**	Day0	109	7(4, 10)	103	6 (3,11)	0.95	
	Day3	109	4 (2, 7)	103	4 (1, 7)	0.41	
	Day7	109	2 (0, 5)	103	3 (1, 7)	0.11	
**APACHEII score in MSAP**,** median (IQR)**	Day0	82	5(3, 9)	73	5 (3, 7)	0.12	
	Day3	82	3 (1, 5)	73	3 (1, 5)	0.43	
	Day7	82	1 (0, 3)	73	2 (0, 7)	0.18	
**APACHEII score in SAP**,** median (IQR)**	Day0	27	11(7, 15)	30	14 (8.75, 16.25)	0.27	
	Day3	27	8 (5, 15)	30	10.5 (5, 15)	0.42	
	Day7	27	5 (2.75, 11)	30	8 (4, 12)	0.11	
**Delta APACHEII score in whole cohort**,** median (IQR)**	Day0-Day3	109	2 (0, 4)	103	2 (1, 4)	0.14	
	Day0-Day7	109	4 (1.5, 7)	103	2 (0, 5)	0.03*	
**Delta APACHEII score in MSAP**,** median (IQR)**	Day0-Day3	82	2 (1, 4)	73	2 (0, 4)	0.318	
	Day0-Day7	82	3 (1, 7)	73	2 (0, 4)	0.01*	
**Delta APACHEII score in SAP**,** median (IQR)**	Day0-Day3	27	3 (0, 6)	30	3 (0, 4)	0.565	
	Day0-Day7	27	5 (3, 8)	30	4 (0, 6)	0.303	
**Modified Marshall score in whole cohort**,** median (IQR)**	Day0	109	1 (0, 3)	103	1 (0, 3)	0.609	
	Day3	109	0 (0, 2)	103	0 (0, 2)	0.435	
	Day7	109	0 (0, 1.25)	103	0 (0, 2)	0.136	
**Modified Marshall score in MSAP**,** median (IQR)**	Day0	82	0 (0, 1)	73	0 (0, 0)	0.312	
	Day3	82	0 (0, 0)	73	0 (0, 0)	0.489	
	Day7	82	0 (0, 0)	73	0 (0, 0)	0.92	
**Modified Marshall score in SAP**,** median (IQR)**	Day0	27	3 (2, 4)	30	3 (2, 5)	0.097	
	Day3	27	2 (2, 4)	30	3 (2, 6)	0.172	
	Day7	27	2 (0, 2)	30	2 (1, 5)	0.09	
**Delta modified Marshall score in whole cohort**,** median (IQR)**	Day0-Day3	109	0 (0, 1)	103	0 (0,0)	0.705	
	Day0-Day7	109	0 (0, 2)	103	0 (0, 1)	0.1723	
**Delta modified Marshall score in MSAP**,** median (IQR)**	Day0-Day3	82	0 (0, 0)	73	0 (0,0)	0.67	
	Day0-Day7	82	0 (0, 1)	73	0 (0, 0)	0.311	
**Delta modified Marshall score in SAP**,** median (IQR)**	Day0-Day3	27	0 (0, 1)	30	0 (-1, 1)	0.337	
	Day0-Day7	27	1 (0, 2)	30	0 (-1, 2)	0.626	
**Balthazar score in whole cohort**,** median (IQR)**	Day0	109	4 (3, 4)	103	4 (3, 4)	0.364	
	Day3	109	4 (3, 4)	103	4 (3, 4)	0.421	
	Day7	109	4 (3, 4)	103	4 (3, 4)	0.337	
**Balthazar score in MSAP**,** median (IQR)**	Day0	82	4 (3, 4)	73	4 (3, 4)	0.223	
	Day3	82	4 (3, 4)	73	4 (3, 4)	0.546	
	Day7	82	4 (3, 4)	73	4 (3, 4)	0.194	
**Balthazar score in SAP**,** median (IQR)**	Day0	27	4 (4, 4)	27	4 (4, 4)	0.576	
	Day3	27	4 (4, 4)	27	4 (4, 4)	0.391	
	Day7	27	4 (4, 4)	27	4 (4, 4)	0.555	
**Delta Balthazar score in whole cohort**,** median (IQR)**	Day0-Day3	109	0 (0, 0)	103	0 (0, 0)	0.932	
	Day0-Day7	109	0 (0, 0)	103	0 (0, 0)	0.942	
**Delta Balthazar score in MSAP**,** median (IQR)**	Day0-Day3	82	0 (0, 0)	73	0 (0, 0)	0.226	
	Day0-Day7	82	0 (0, 0)	73	0 (0, 0)	0.641	
**Delta Balthazar score in SAP**,** median (IQR)**	Day0-Day3	27	0 (0, 0)	30	0 (0, 0)	0.572	
	Day0-Day7	27	0 (0, 0)	30	0 (0, 0)	0.205	

(a) IQR, interquartile range; (b) RRT, renal replacement therapy; (c) MV, mechanical ventilation. (d) MSAP, moderately severe acute pancreatitis; (e) SAP, severe acute pancreatitis; (f) HDIVC, high dose intravenous vitamin C. Organ failure was defined as a modified Marshall score of 2 or more in one or more systems, including respiratory, renal, and cardiovascular. MV-free duration was defined as the number of hours a patient was not on mechanical ventilation, and RRT-free duration was defined similarly. The Sequential Organ Failure Assessment (SOFA) score ranges from 0 to 24, with higher scores indicating greater severity of organ dysfunction. The Acute Physiology and Chronic Health Evaluation II score (APACHE II) ranges from 0 to 71, with higher scores indicating a greater risk of hospital death. The Balthazar score ranges from 0 to 4, with higher scores indicating more inflammatory exudates around the pancreas. The modified Marshall score ranges from 0 to 4 in the respiratory, renal, and cardiovascular systems separately; a score of 2 or more in any system defines the presence of organ failure

### Sensitivity analysis

Of the 212 enrolled patients, 2 were excluded because of protocol deviations, leaving 210 patients in the per-protocol analysis set. There were 2 deaths in the HDIVC group (1.9%) and 3 in the placebo group (2.8%). The unadjusted odds ratio (OR) for death in the HDIVC group was 0.622 at 28 days (95% CI, 0.101–3.804; *p* = 0.608).

### Adverse events

Patients were followed up for adverse events. Crystalluria was found in 2 patients in the HDIVC group during intervention. The study medication was continued, and no serious events occurred. The patient’s crystalluria disappeared quickly after 7 days of intervention. There were no reported other study-related adverse events or severe adverse events during the trial.

## Discussion

In this randomized, placebo-controlled clinical trial involving adults with MSAP and SAP, treatment with HDIVC did not result in a decrease in 28-day mortality. Subgroup analysis revealed that HDIVC treatment was associated with a significant reduction in CRP levels, fluid retention, and APACHE II scores in a subgroup of MSAP patients. However, it is important to note that these findings should be considered exploratory.

The trial was terminated earlier than anticipated due to a lower than expected 28-day mortality rate. This unexpected outcome may have been influenced by the high proportion of enrolled MSAP patients in each group (75.2% and 70.9%) and the declining mortality in SAP patient in our center. Previous reports indicate that the mortality rate in MSAP patients is approximately 1%, significantly lower than the 20-40% mortality rate observed in SAP patients [19, 20]. The 28-day mortality rates were 2.8% (3/109) in the HDIVC group and 3.9% (4/103) in the control group, showing no significant difference (*p* = 0.939). This outcome aligns with findings from two previously published randomized controlled trials on SAP [10, 11]. However, it should be mentioned that the definition of SAP was not comparable between these studies. Moreover, in previous reports, vitamin C was combined with other antioxidants, such as selenium, N-acetyl cysteine, vitamin A or vitamin E, and the dose of vitamin C was relatively low (1 g/d ~ 2 g/d, iv).

In this study, we found that the HDIVC group showed a significant reduction of CRP levels during the intervention period from Day 0 to Day 3 and from Day 0 to Day 7. However, further analysis of subgroups revealed that the reduction of CRP was significant only in MSAP patients, not in SAP patients. This finding is consistent with a study on moderate COVID-19 patients treated with intravenous vitamin C, which also showed a decrease in CRP levels [21]. Conversely, high dose intravenous vitamin C did not affect CRP levels in sepsis with severe acute respiratory failure [15]. Preclinical research has shown that vitamin C may help reduce oxidative stress in acute pancreatitis [8]. This effect was also observed in septic models [22]. Additionally, since the HDIVC group had higher baseline CRP levels, we reanalyzed the proportional decline in CRP and found a significant difference between the two groups from Day 0 to Day 3, but not from Day 0 to Day 7. This suggests that HDIVC may be more effective at lowering CRP during the early phase (day 0 to day 3), when CRP levels are still relatively high. By Day 7, the inflammatory response in SAP had diminished, and CRP levels in both groups were lower, with the difference in absolute values likely reflecting baseline differences rather than HDIVC’s effect. This finding suggests that HDIVC may accelerate the clearance of oxidative mediators during moderate inflammatory responses but does not appear to have a significant impact on severe or minor inflammatory surges.

Fluid resuscitation is a crucial therapy in the early stages of acute pancreatitis. In this study, we treated enrolled patients with controlled fluid resuscitation [18] which include two phases. Initially, aggressive fluid resuscitation at 5–10 ml/kg is administered for blood volume expansion, and once resuscitation targets such as heart rate, urine output, hematocrit, and mean arterial pressure are achieved, a shift to restricted fluid resuscitation for adjustment of body fluid distribution. During the restricted fluid resuscitation phase, the use of higher volume of colloids ( mainly albumin) and diuretics (Supplementary Table 8), or Renal Replacement Therapy (RRT), aims to ensure that the output exceeds the input, thereby reducing fluid retention in the third space. Fluid retention is a crucial prognostic indicator during the fluid resuscitation phase. This is because excessive fluid retention is closely with fatal complication including abdominal compartment syndrome, acute respiratory failure, and infected pancreatic necrosis [23]. In this study, the HDIVC group showed a significant reduction in fluid retention compared to the control group among MSAP patients. Similar findings have been reported in burn patients [24]. This phenomenon may be explained by the protective effect of HDIVC on the endothelial vascular barrier damaged by oxidative mediators. Additionally, vitamin C may help stabilize circulation as a cofactor for endogenous catecholamine oxidase and may reduce the fluid resuscitation volume [25].

The APACHE II score is a well-established severity scoring system used in critical care. Although it was not specifically designed for acute pancreatitis, it might be useful in monitoring disease progression and response to therapy [26]. In this study, the change in APACHE II score from Day0 to Day7 was significantly greater in HDIVC group compared to the Control group in both the entire cohort and MSAP subgroup. This finding may be attributed to the decrease in CRP levels and reduction in fluid retention.

Our study demonstrated HDIVC provided benefits for patients with MSAP but not for those with SAP. This finding is partially supported by a small sample size study (*n* = 84) by Du et al. [12] where they observed that treatment with HDIVC (10 g/day) was associated with a higher cure rate, lower complication rate, and shorter hospital stay. Notably, more than 83% of the patients included in their study had MSAP. HDIVC may have a beneficial effect on mitigating injury in patients with mild severity of acute pancreatitis, but its impact on more severe cases remains unclear.

This study has several limitations. Firstly, the premature termination due to low participant mortality resulted in an underpowered sample size, limiting the detection of clinically significant 28-day mortality differences. Secondly, the initial severity of severe acute pancreatitis (SAP), assessed by the SOFA score (median 4 vs. 6, *P* = 0.002), was unequal between groups, potentially skewing the shorter ICU stay and reduced costs observed in the HDIVC group (sTable1). Thirdly, the changes in CRP and fluid retention were not pre-specified in the original protocol and were added later as exploratory outcomes, so their interpretation should be cautious. Fourthly, the effect observed for these outcomes varied across different analyses and subgroups, suggesting that the benefits may not be consistent in all patient groups. Fifthly, while APACHE II is not specifically designed for acute pancreatitis, it may not be the most reliable tool for monitoring changes in MSAP and SAP patients. Therefore, a multicenter randomized trial is needed to definitively assess the impact of HDIVC.

## Conclusions

HDIVC did not significantly reduce the risk of death within 28 days among patients with MSAP and SAP. While it demonstrated a potential benefit in reducing CRP levels, fluid retention, and APACHE II scores in MSAP patients, these changes may not be directly attributable to the study drug. Furthermore, no similar changes were observed in SAP patients. Anyway, these findings should be regarded exploratory and a multicenter randomized clinical trial is warranted in future.

## Electronic supplementary material

Below is the link to the electronic supplementary material.

Supplementary Material 1

Supplementary Material 2

Supplementary Material 3

Supplementary Material 4

## Data Availability

The data sets used and analyzed are available from the corresponding author on reasonable request.

## Abbreviations

AP: Acute Pancreatitis
MSAP: Moderately Severe Acute Pancreatitis
SAP: Severe Acute Pancreatitis
HDIVC: High-Dose Intravenous Vitamin C
CRP: C-Reactive Protein
SIRS: Systemic Inflammatory Response Syndrome
SOFA: Sequential Organ Failure Assessment
APACHE II: Acute Physiology and Chronic Health Evaluation II
MODS: Multiple Organ Dysfunction Syndrome
ICU: Intensive Care Unit
RRT: Renal Replacement Therapy
MV: Mechanical Ventilation
APS: Acute Physiology Score
CHS: Chronic Health Score
ERCP: Endoscopic Retrograde Cholangiopancreatography
IQR: Interquartile Range
ROS: Reactive Oxygen Species
CITRIS: ALI-Vitamin C Infusion for Treatment in Sepsis-Induced Acute Lung Injury
ChiCTR: Chinese Clinical Trial Registry
MAP: Mild Acute Pancreatitis
UDPN: Unblinded Drug Preparing Nurse
BMI: Body Mass Index
HCT: Hematocrit
ANC: Acute Necrosis Collection
APFC: Acute Peripancreatic Fluid Collection
