# Supplementary material for: High-dose intravenous vitamin C reduce C-reactive protein levels, fluid retention, and APACHE II scores in patients with moderately severe acute pancreatitis: a prospective, randomized, double-blinded, placebo-controlled study
Source: Ann Intensive Care. 2025 Mar 17;15:30. doi: 10.1186/s13613-025-01437-z (PMC11911288; doi:10.1186/s13613-025-01437-z)
Supplement: Supplementary file 1 — Supplementary Material 1 [file 13613_2025_1437_MOESM1_ESM.docx]

**专家咨询意见**

我院自2019年9月1日起开展了一项关于大剂量维生素C治疗中重度急性胰腺炎的双盲、单中心随机对照临床研究。原计划于2023年12月31日结束，预计纳入418名符合标准的胰腺炎患者，以28天死亡率为主要终点指标进行计算。

截至2023年6月19日，已入组患者212人，包括中度重症急性胰腺炎（MSAP）155例和重症急性胰腺炎（SAP）57例。然而在试验过程中，我们发现本中心重症急性胰腺炎患者的28天死亡率为12%（7/57），中度重症急性胰腺炎28天死亡率为0%（0/155），远低于计算样本量时的估计水平（估算样本量方法：MSAP 和SAP死亡率35%，而治疗组死亡率下降10%）。这可能导致样本量估计有误，统计效能不足，使得研究无法得出预先设想的结论。因此，我们于2023年6月19日召开外部专家咨询线上会议，讨论是否需提前中止本研究。

出席此次会议的专家包括：海军军医大学长海医院消化内科杜亦奇主任，四川大学华西医院急诊科曹珏主任，复旦大学公共卫生学院生物统计教研室赵耐青教授，南昌大学附属第一医院消化内科祝荫主任。

经过深入讨论，与会专家一致认为：

一、由于当前28天死亡率远低于预期，导致样本量估计有误，即使按预期样本量完成入组，也无法验证研究假设，因此建议中止该研究。

二、对于已收集的数据，建议进行事后分析，为后续类似研究提供更多的信息。

三、考虑到各中心医疗水平差异可能导致死亡率偏倚，建议后续开展多中心研究以消除此偏倚。

综合上述专家的意见，终止该研究并针对现有数据进行分析。

**
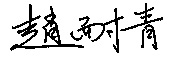
专家签名：
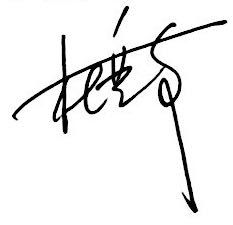

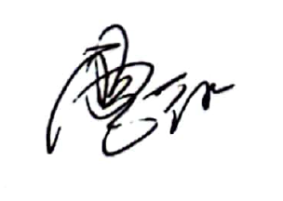

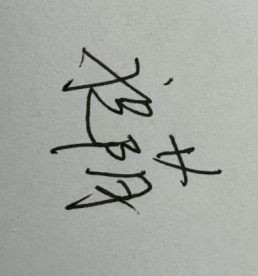
**

**日期：**

**Resolution of expert consultation**

Our hospital has conducted a double-blind, single-center, randomized controlled clinical study on the treatment of moderate to severe acute pancreatitis with high-dose vitamin C since September 1, 2019. The original plan was to end on December 31, 2023, with an estimated enrollment of 418 patients with pancreatitis who meet the criteria, using 28-day mortality as the primary endpoint.

As of June 19, 2023, 212 patients had been enrolled, including 155 cases of moderately severe acute pancreatitis (MSAP) and 57 cases of severe acute pancreatitis (SAP). However, during the course of the trial, we found that the 28-day mortality rate for patients with severe acute pancreatitis and moderately severe acute pancreatitis at our center were 12% (7/57) and 0%(0/155) respectively, which was much lower than the estimated level when calculating the sample size (estimation method: MSAP and SAP mortality rate of 35%, with a 10% reduction in mortality rate in the treatment group). This may have led to an error in sample size estimation and insufficient statistical power, making it impossible for the study to reach the expected conclusion.

Therefore, on June 19, 2023, we held an online meeting of expert consultation to discuss whether to terminate the study early. The experts attending the meeting included: Director Du Yiqi from the Digestive Disease Department of Changhai Hospital of Naval Medical University, Director Cao Yue from the Emergency Department of West China Hospital of Sichuan University, Professor Zhao Naiqing from the Statistics Teaching and Research Section of the School of Public Health at Fudan University, and Director Zhu Yin from the Digestive Disease Department of the First Affiliated Hospital of Nanchang University. After deep discussion, the experts unanimously agreed that:

First, due to the current 28-day mortality rate being far lower than expected, leading to an error in sample size estimation, even if the enrollment is completed according to the expected sample size, it will not be possible to verify the research hypothesis, so it is recommended to terminate the study. Second, for the data already collected, it is recommended to conduct post-hoc analysis to provide more information for similar studies in the future. Third, considering that differences in medical levels among centers may lead to mortality bias, it is recommended to conduct multicenter studies in the future to eliminate this bias.

Based on the opinions of the experts, the study was terminated and an analysis was conducted on the existing data.

**Expert signatures:
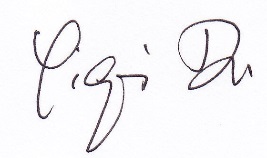

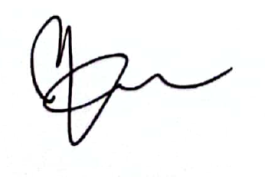

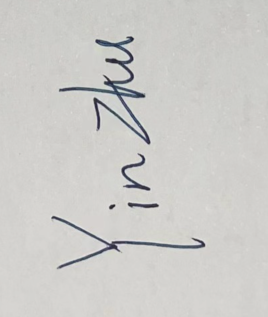
**
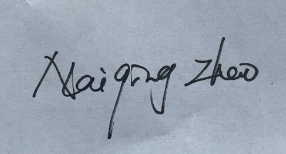


**Date:**
