# Supplementary figures and images for: High-dose intravenous vitamin C reduce C-reactive protein levels, fluid retention, and APACHE II scores in patients with moderately severe acute pancreatitis: a prospective, randomized, double-blinded, placebo-controlled study
Source: Ann Intensive Care. 2025 Mar 17;15:30. doi: 10.1186/s13613-025-01437-z (PMC11911288; doi:10.1186/s13613-025-01437-z)

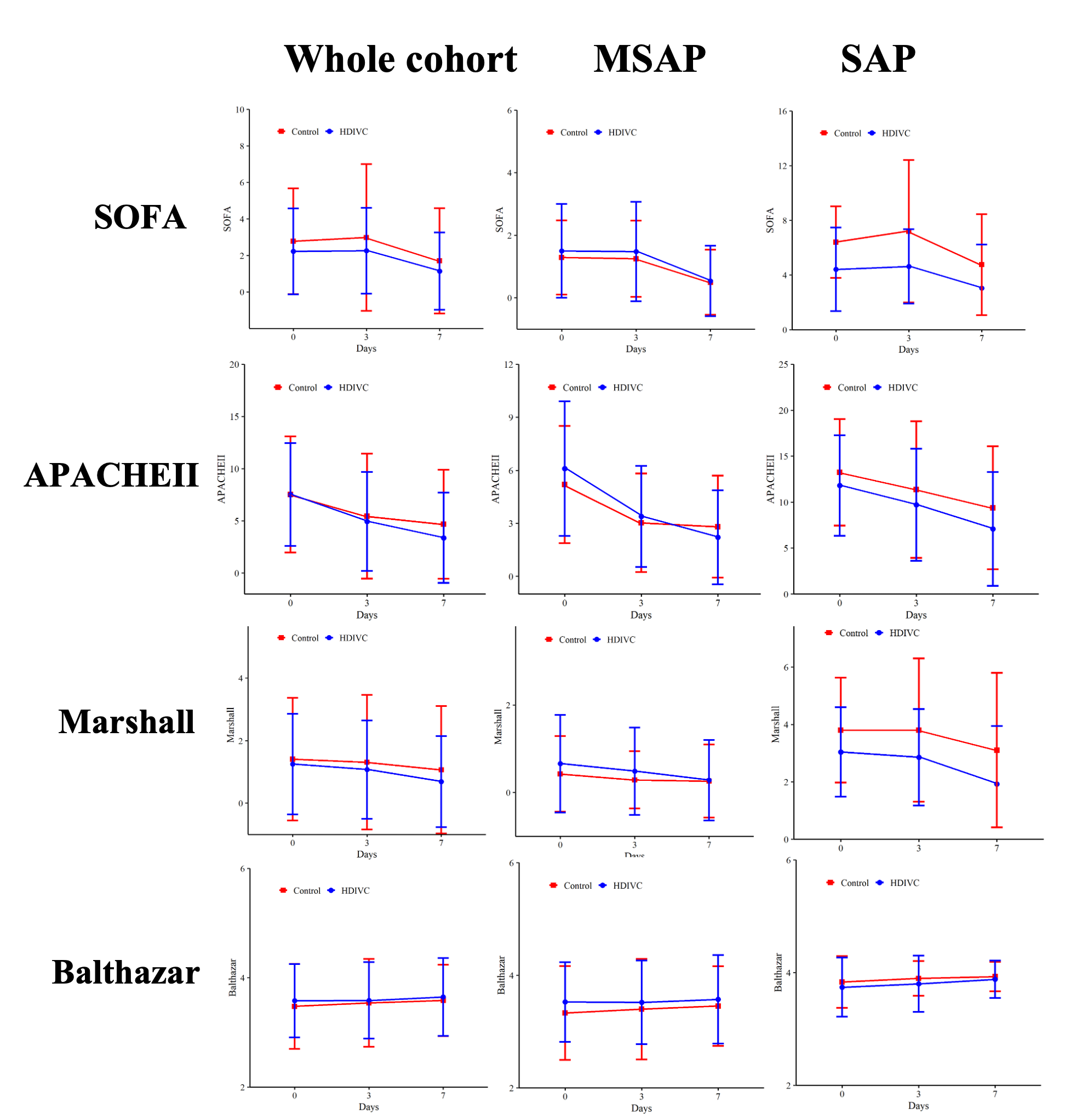

Supplement: Supplementary file 2 — Supplementary Material 2 [file 13613_2025_1437_MOESM2_ESM.png]
