# Supplementary material for: High-dose intravenous vitamin C reduce C-reactive protein levels, fluid retention, and APACHE II scores in patients with moderately severe acute pancreatitis: a prospective, randomized, double-blinded, placebo-controlled study
Source: Ann Intensive Care. 2025 Mar 17;15:30. doi: 10.1186/s13613-025-01437-z (PMC11911288; doi:10.1186/s13613-025-01437-z)
Supplement: Supplementary file 3 — Supplementary Material 3 [file 13613_2025_1437_MOESM3_ESM.docx]

**Supplementary Figure 1 Dynamics of scores on Day0, Day3 and Day7**

SOFA, Sequential Organ Failure Assessment score; APACHE II, The Acute Physiology and Chronic Health Evaluation II ; MSAP, moderately severe acute pancreatitis; SAP, severe acute pancreatitis; HDIVC, high dose intravenous vitamin C
