# Supplementary material for: High-dose intravenous vitamin C reduce C-reactive protein levels, fluid retention, and APACHE II scores in patients with moderately severe acute pancreatitis: a prospective, randomized, double-blinded, placebo-controlled study
Source: Ann Intensive Care. 2025 Mar 17;15:30. doi: 10.1186/s13613-025-01437-z (PMC11911288; doi:10.1186/s13613-025-01437-z)
Supplement: Supplementary file 4 — Supplementary Material 4 [file 13613_2025_1437_MOESM4_ESM.docx]

**Supplementary Table 1: Pre-enrollment treatments for patients transferred from other facilities**

| **Variables** | **HDIVC (n=23)** | **Control (n=27)** | **P-value** |
| --- | --- | --- | --- |
| **Fluid resuscitation, NO.(%)** | 23（100%） | 27（100%） | 1.0 |
| **Fasting, NO.(%)** | 23（100%） | 27（100%） | 1.0 |
| **Pancreatic enzyme inhibitors, NO.(%)** | 11（47.8%） | 13（48.1%） | 1.0 |
| **Antibiotics, NO.(%)** | 17（73.9%） | 20（74.1%） | 1.0 |
| **Gastrointestinal decompression, NO.(%)** | 10（43.5%） | 6（22.2%） | 0.193 |
| **PPI, NO.(%)** | 14（60.9%） | 15（55.6%） | 0.927 |

**Supplementary Table 2 Diagnostic criteria for MSAP**

|  | **HDIVC (n=82)** | **Control (n=73)** | **P-value** |
| --- | --- | --- | --- |
| **Local complication** |  |  | 0.567 |
| **APFC, NO. (%)** | 55 (67.1%) | 53 (72.6%) |  |
| **ANC, NO. (%)** | 27 (32.9%) | 20 (27.4%) |  |
| **Organ failure** |  |  |  |
| **Cardiovascular system, NO. (%)** | 0 | 0 | / |
| **Respiratory system, NO. (%)** | 20 (24.4%) | 9 (12.3%) | 0.086 |
| **Renal system, NO. (%)** | 8 (9.8%) | 8 (11.0%) | 1.0 |

MSAP, moderate severe acute pancreatitis; APFC, acute peripancreatic fluid collection; ANC, acute necrosis collection; HDIVC, high dose intravenous vitamin C

**Supplementary Table 3 Proportional decline of CRP.**

| **Variable** | **HDIVC** | **Control** | **P-value** | |  |
| --- | --- | --- | --- | --- | --- |
| **Total (n = 212)** |  |  | |  | |
| **Day0-Day3** | 29.2 (9.7, 49.1) | 16.6 (-19.2, 34.6) | | 0.003* | |
| **Day0-Day7** | 69.8 (45.1, 87.2) | 60.0 (24.2, 85.0) | | 0.363 | |
| **MSAP (n = 155)** |  |  | |  | |
| **Day0-Day3** | 29.5 (10.1, 49.0) | 16.0 (-21.8, 37.6) | | 0.005* | |
| **Day0-Day7** | 73.4 (50.0, 88.2) | 69.4 (38.7, 87.2) | | 0.108 | |
| **SAP (n= 57)** |  |  | |  | |
| **Day0-Day3** | 22.7 (9.0, 48.4) | 21.8 (3.6, 30.9) | | 0.371 | |
| **Day0-Day7** | 47.3 (29.6, 74.0) | 40.9 (12.6, 67.7) | | 0.486 | |
| Differences are presented as median (IQR). CRP, C-reactive protein; MSAP, moderate severe acute pancreatitis; HDIVC, high dose intravenous vitamin C. *P <0.05. | | | | | |

**Supplementary Table 4 Effect of HDIVC on components of APACHEII score in whole cohort population**

| **Variable** | **HDIVC** | **Control** | **P-value** | |  |
| --- | --- | --- | --- | --- | --- |
| **APS** |  |  | |  | |
| **Day0-Day3** | 2 (1, 4) | 2 (0, 4) | | 0.391 | |
| **Day0-Day7** | 4 (2, 7) | 2 (0, 5) | | 0.012* | |
| **CHS** |  |  | |  | |
| **Day0-Day3** | 0 (0, 0) | 0 (0, 0) | | 1 | |
| **Day0-Day7** | 0 (0, 0) | 0 (0, 0) | | 1 | |
| **APS plus CHS** |  |  | |  | |
| **Day0-Day3** | 2 (1, 4) | 2 (0, 4) | | 0.391 | |
| **Day0-Day7** | 4 (2, 7) | 2 (0, 5) | | 0.012* | |
| Differences are presented as median (IQR). APS , acute physiology score; CHS, chronic health score; HDIVC, high dose intravenous vitamin C  *P <0.05. | | | | | |

**Supplementary Table 5: Effect of HDIVC on components of APACHEII score in MSAP group**

| **Variable** | **HDIVC** | **Control** | **P-value** | |  |
| --- | --- | --- | --- | --- | --- |
| **APS** |  |  | |  | |
| **Day0-Day3** | 2 (1, 4) | 2 (0, 4) | | 0.291 | |
| **Day0-Day7** | 3 (1, 7) | 2 (0, 4) | | 0.013* | |
| **CPS** |  |  | |  | |
| **Day0-Day3** | 0 (0, 0) | 0 (0, 0) | | 1 | |
| **Day0-Day7** | 0 (0, 0) | 0 (0, 0) | | 1 | |
| **APS+CHS** |  |  | |  | |
| **Day0-Day3** | 2 (1, 4) | 2 (0, 4) | | 0.291 | |
| **Day0-Day7** | 3 (1, 7) | 2 (0, 4) | | 0.013* | |
| Differences are presented as median (IQR). APS , acute physiology score; CHS, chronic health score; HDIVC, high dose intravenous vitamin C.  *P <0.05. | | | | | |

**Supplementary Table 6 Effect of HDIVC on components of APACHEII score in SAP group**

| **Variable** | **HDIVC** | **Control** | **P-value** | |  |
| --- | --- | --- | --- | --- | --- |
| **APS** |  |  | |  | |
| **Day0-Day3** | 3 (0, 6) | 3 (-1, 4) | | 0.877 | |
| **Day0-Day7** | 4 (3, 8) | 4 (0, 6) | | 0.303 | |
| **CHS** |  |  | |  | |
| **Day0-Day3** | 0 (0, 0) | 0 (0, 0) | | 1 | |
| **Day0-Day7** | 0 (0, 0) | 0 (0, 0) | | 1 | |
| **APS+CHS** |  |  | |  | |
| **Day0-Day3** | 3 (0, 6) | 3 (-1, 4) | | 0.877 | |
| **Day0-Day7** | 4 (3, 8) | 4 (0, 6) | | 0.303 | |
| Differences are presented as median (IQR). APS , acute physiology score; CHS, chronic health score; HDIVC, high dose intravenous vitamin C.  *P <0.05. | | | | | |

**Supplementary table 7 Effect of HDIVC on other secondary outcomes**

| **Variables** | **HDIVC^e^**  **(n = 109)** | **Control**  **(n = 103)** | **P-value** | | |
| --- | --- | --- | --- | --- | --- |
| **90 Day mortality, No. (%)** | 5 (4.59%) | 4 (3.88%) | 0.99 | |  |
| **IPN^a^ occurrence, No. (%)** | 16(14.68%) | 22 (21.36%) | 0.22 | |  |
| **ICU length of whole cohort，median (IQR^b^), d** | 15 (8, 26) | 20 (11, 33) | **0.03*** | |  |
| **ICU length of SAP^c^，median (IQR), d** | 20 (11, 35) | 31 (22, 61) | **0.02*** | |  |
| **ICU length of MSAP^d^，median (IQR), d** | 13 (7, 20) | 11 (7, 17.5) | 0.59 | |  |
| **Cost of whole cohort, median (IQR), ¥** | 65760 (44810, 104692) | 68750 (41461, 128307) | 0.09 | |  |
| **Cost of MSAP median (IQR), ¥** | 52396 (37924, 77953) | 57507 (37432, 75283) | 0.77 | |  |
| **Cost of SAP, median (IQR), ¥** | 118412 (76978, 203131) | 216369 (112269,460373) | **0.02*** | |  |
| a. IPN, infected pancreatic necrosis; b. IQR, interquartile range; c. SAP, severe acute pancreatitis; d. MSAP, moderately severe acute pancreatitis; e. HDIVC, high dose intravenous vitamin C. *: P<0.05 | | | |  |  |

**Supplementary table 8 Usage of diuretics in the whole cohort, MSAP and SAP**

| **Variable** | **HDIVC** | | | | **Control** | | **P-value** | |
| --- | --- | --- | --- | --- | --- | --- | --- | --- |
| **whole cohort** | |  | |  | |  | |  |
| **Use of furosemide (%)** | | 92/109 (84.4) | | 87/103 (84.5) | | 0.99 | |  |
| **Average furosemide doses (mg/day)** | | 12.0 (4.0-22.0) | | 13.0 (4.0-21.2) | | 0.92 | |  |
| **SAP** | |  | |  | |  | |  |
| **Use of furosemide (%)** | | 24/27 (88.9) | | 23/30 (76.7) | | 0.388 | |  |
| **Average furosemide doses (mg/day)** | | 27.0 (9.0-36.8) | | 24.9 (12.4-33.6) | | 0.773 | |  |
| **MSAP** | |  | |  | |  | |  |
| **Use of furosemide (%)** | | | 68/82 (82.9) | 64/73 (87.7) | | 0.407 | |  |
| **Average furosemide doses (mg/day)** | | | 12.0 (4.0-16.4) | 10.0 (4.0-15.0) | | 0.747 | |  |
| SAP, severe acute pancreatitis; MSAP, moderate severe acute pancreatitis; HDIVC, high dose intravenous vitamin C | | | | | | | |  |
